# Reducing hand recontamination of healthcare workers during COVID-19

**DOI:** 10.1017/ice.2020.111

**Published:** 2020-04-06

**Authors:** Giorgia Gon, Stephanie Dancer, Robert Dreibelbis, Wendy J. Graham, Claire Kilpatrick

**Affiliations:** 1 Department of Infectious Disease Epidemiology, London School of Hygiene and Tropical Medicine, London, United Kingdom; 2 Department of Microbiology, Hairmyres Hospital & School of Applied Sciences, Edinburgh Napier University, Edinburgh, United Kingdom; 3 Department of Disease Control, London School of Hygiene and Tropical Medicine, London, United Kingdom; 4 World Health Organization Consultant, Glasgow, United Kingdom

*To the Editor*—Worldwide, the response to the COVID-19 pandemic requires hand hygiene compliance by everyone, as highlighted in the WHO #SafeHands campaign and numerous documents. Hand hygiene is particularly critical for frontline healthcare workers (HCWs) who are overstretched and for whom this key routine task must be easy to complete and effective.^1^ However, a neglected aspect of hand hygiene, even in the absence of a global pandemic, is the risk of touching surfaces or objects that could recontaminate hands after hand rubbing or washing, whether gloves are worn or not. Infection prevention is key during this pandemic, and reducing hand recontamination is important to ensuring patient and HCW safety at all times.

Avoiding recontamination is implicit in the WHO Hand Hygiene guidelines for health facilities.^2^ Failure to comply with hand hygiene can result from not washing or rubbing hands at the right time or from subsequent hand or glove recontamination. In a recent study in Tanzania during which 781 hand hygiene indications were observed, approximately half of the times when birth attendants rubbed or washed their hands, they then recontaminated their hands on potentially unclean surfaces before performing an aseptic procedure.^3^ Similar findings come from obstetric wards in Nigeria and Ghana.^4,5^ Recontamination is not only a problem in low-income settings. A US study demonstrated microbiological recontamination of hands at the point of care despite high levels of self-reported hand hygiene compliance.^6^ Reports from the United Kingdom and Australia show that HCWs touch privacy curtains between hand hygiene and touching a patient.^7^

The Tanzanian study also suggested that hand rubbing or washing and glove recontamination are underpinned by different behavioral determinants.^8^ Without targeting these 2 behaviors separately, hand hygiene initiatives during this pandemic may be undermined.

HCWs are able to prioritize patient needs when providing routine care. However, the COVID-19 pandemic has introduced significant uncertainty into the care environment and thus workflow, including timing of necessary procedures, anticipating and managing patient volumes, and rapidly evolving guidelines on patient management. During this crisis, hand hygiene, along with other infection control activities, may be compromised, not because it is not a priority but rather because staff may be too busy or uncertain on how to implement hand hygiene in this outbreak setting. In their ethnography of infection prevention in Australia, Hor et al^9^ state that understanding the “boundaries of what is clean” is not straightforward in hospital departments and that HCWs have different perceptions over whether certain surfaces could potentially lead to cross transmission. Recontamination may be an indication that staff fail to understand the definition of the WHO hand hygiene recommendations or how those apply in rapidly changing healthcare settings.^3^

**Fig. 1.**
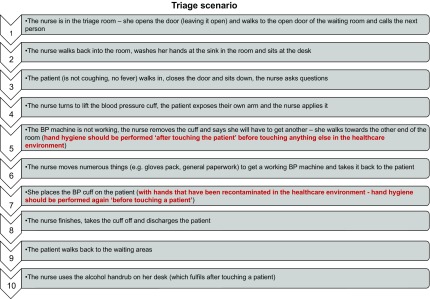
Example of hand recontamination during a triage scenario.

An understanding of surfaces that are safe to touch depend upon assumptions about appropriate cleaning of surfaces, cleaning frequencies, established methods, and sufficient trained cleaning staff. In spite of amazing efforts from all staff, including environmental cleaning staff, standards are not always optimal in the United Kingdom, as in many other countries.^10^ Surface contamination played a plausible role in SARS, MERS, and pandemic influenza transmission in healthcare settings. Emerging evidence suggests that the virus responsible for the current pandemic (SARS-CoV-2) can survive on common surfaces for days, but viral demographics and characteristics have yet to be sufficiently studied.^11^ Recontamination of hands is a consequence and a source of poor surface cleanliness (Fig. 1, Steps 5 and 7).

We call for greater attention to the risk from hand recontamination and the opportunity for its prevention through empowering HCWs and strengthening cleaning of the care environment. For those managing COVID-19 cases, these actions will improve their own and coworkers’ safety as well as that of all patients and visitors.

Like so much in the COVID-19 response, behavior change plays a key part. Behavior change needs to be tailored and targeted. Following Michie’s principles for behavior change during the COVID-19 pandemic,^1^ we recommend the following:

1. A mental model: Training, monitoring, and feedback should include clear guidance for understanding the “boundaries of what surfaces are clean” with directions on what HCWs can and cannot touch within the patient zone (see the example in Fig. 1), in relation to hand hygiene, especially before a clean or aseptic procedure.
2. Social norms: Managers and their colleagues should lead by example by demonstrating appropriate hand hygiene including avoiding recontamination. Hand hygiene protocols should be followed by everyone involved in patient care.
3. Emotion: The importance of recontamination in patient and HCW safety needs to be clearly emphasized.
4. Replace the behavior to stop the habit: “Keep hands off unsafe surfaces” rather than “do not touch unsafe surfaces.”
5. Make it easy: Create a user-friendly environment that facilitates hand hygiene and reduces opportunities for recontamination. The environment needs to account for the workflow for patient management, allowing for minimal opportunities to recontamination when collecting equipment or moving between patients. The environment should also include appropriate cues to remind and trigger hand hygiene, such as strategic placement of handrub dispensers.
